# 360-Degree laser retinopexy in primary vitrectomy for rhegmatogenous retinal detachment: factors associated with its use and impact on surgical outcomes

**DOI:** 10.1186/s40942-022-00377-1

**Published:** 2022-04-06

**Authors:** Matthew C. Peters, Alexander Murray-Douglass, Joseph Park, Sean S. H. Cheng, Anil K. Sharma, Abhishek Sharma, Kevin W. Vandeleur, Lawrence R. Lee, Thomas P. Moloney

**Affiliations:** Ophthalmology Department, Royal Brisbane and Women’s Hospital, Level 8, Ned Hanlon Building, Butterfield Street, Herston, QLD 4029 Australia

**Keywords:** 360 Laser retinopexy, Retinopexy, Retinal detachment

## Abstract

**Background:**

To determine patient and surgical factors associated with the use of 360-degree laser retinopexy during primary pars plana vitrectomy (PPV) ± scleral buckle (SB) for rhegmatogenous retinal detachment (RRD) and its impact on surgical outcomes.

**Methods:**

Patients who underwent PPV ± SB for repair of non-complex RRD at a single centre were included in this retrospective study. The primary outcome was single surgery anatomical success (SSAS). Secondary outcomes included visual acuity, epiretinal membrane formation, the presence of cystoid macular oedema, tonic pupil and corneal epithelial defects. Multiple logistic regression and multivariate regression was used.

**Results:**

The study included 192 cases, of which 130 received 360-degree laser. Worse preoperative logMAR visual acuity (P = 0.009), male sex (P = 0.060), higher PVR grades, supplemental SB (P = 0.0468) and silicone oil/C_3_F_8_ tamponade (P < 0.0001) were associated with 360-degree laser use. No significant associations between 360-degree laser and SSAS (P = 0.079), final logMAR visual acuity (P = 0.0623), ERM development (P = 0.8208), postoperative CMO (P = 0.5946), tonic pupil (P > 0.9999) or corneal epithelial defects (P = N/A) were found.

**Conclusions:**

360-degree laser retinopexy during primary PPV ± SB for RRD was associated with more complex cases and more extensive operations. Even when accounting for this, there was no difference in surgical outcomes or complication rates.

## Background

Rhegmatogenous retinal detachment (RRD) is a vision-threatening condition caused by tractional forces of the vitreous onto the retina leading to full-thickness retinal tears and accumulation of fluid in the subretinal space [1]. As a consequence of the neurosensory retina separating from the retinal pigment epithelium (RPE), the outer retina loses its choroidal blood supply and becomes ischaemic [1]. This can lead to significant visual morbidity, particularly if there is extension into the macula [1, 2]. Therefore, timely and successful treatment of RRD is crucial for improved visual prognosis [3].

Successful surgical management of RRD requires effective treatment of retinal breaks and relief of vitreoretinal traction [4, 5]. Techniques in use include pneumatic retinopexy (PR), pars plana vitrectomy (PPV), scleral buckling (SB) and combination PPV and SB [3]. Traditionally, SB was considered the procedure of choice for primary RRD [6]. However, PPV continues to increase in popularity as a first-line procedure [3, 6]. The major advantage of PPV is the potential for improved visualisation of the retinal periphery, allowing increased identification of retinal breaks [6]. Disadvantages include the risk of new retinal breaks, cataract formation and elevation of intraocular pressure [6].

Single surgery anatomical success (SSAS) is a key outcome in primary RRD repair as the risk of recurrent detachment and poor functional outcome increases with subsequent procedures [7]. Missed retinal breaks, new retinal break formation, opening of old retinal breaks and proliferative vitreoretinopathy have been identified as major causes of retinal re-detachment [4, 8]. One method proposed to reduce re-detachment rates in patients undergoing PPV is 360-degree prophylactic endolaser photocoagulation. Theoretically, application of 360-degree laser creates a chorioretinal scar that may wall off subretinal fluid conduits from anterior to posterior retinal areas and cover any missed breaks [4, 9]. However, studies that have looked at its use in primary PPV for RRD have yielded conflicting results [4, 10–14].

The current study evaluates of the effect of 360-degree laser on the rate of SSAS following primary PPV with or without SB for RRD. The study also investigates the impact of 360-degree laser on visual outcome and incidence of common complications.

## Methods

Ethics approval for this study was waived by The Royal Brisbane and Women’s Hospital Human Research Ethics Committee (HREC) (EC00172) as it does not meet the criteria for research and was approved as a quality assurance study. This study was conducted in accordance with the Declaration of Helsinki. Verbal informed consent was obtained from all subjects before the study.

### Clinical data collection

Patients who had PPV with or without SB for repair of RRD at Royal Brisbane and Women’s Hospital (RBWH) from June 2017 to December 2020 were captured in the database. Recurrent detachments, proliferative diabetic retinopathy and retinal detachments secondary to trauma, were excluded from the dataset. Patients with fewer than 3 months of postoperative follow-up or who were lost to follow-up (either to RBWH or any Queensland Health facility) were also excluded.

Detailed demographic, preoperative, intraoperative and postoperative follow-up variables were collected from RBWH data using the Ophthalmic Research Institute of Australia Retinal Detachment database. These variables were confirmed against the scanned medical records on Queensland Health’s integrated electronic medical record (ieMR) system. The primary outcome considered was SSAS. SSAS was defined as retinal attachment with no tamponade present and no presence of significant subretinal fluid at 3 months postoperatively. Secondary outcomes included logarithm of the minimum angle of resolution (logMAR) visual acuity, epiretinal membrane (ERM) formation, the presence of cystoid macular oedema (CMO), tonic pupil and corneal epithelial defects at 3 months postoperative follow-up.

### Surgical considerations

Surgery was undertaken by one of the authors with a standard 25-gauge vitrectomy using the Alcon Constellation System. No case had cataract surgery/lensectomy at the time of the primary RD repair. Laser was the only type of retinopexy used in these cases. It was at the surgeon’s discretion as to whether a scleral buckle was to be used adjunctively, 360-degree laser retinopexy or limited laser retinopexy was used and the type of tamponade. If silicone oil was used 5700 centistoke oil was used exclusively.

The indication for 360-degree laser was again at the surgeon’s discretion but included prophylaxis against missed or new breaks, extensive breaks or detachment, and in giant retinal tear related RRD. In general 360-degree laser retinopexy was to a light grey burn and was applied in a milder fashion compared to laser limited to around retinal breaks or a retinotomy.

### Statistical analysis

Multiple logistic regression modelling was performed with the use of 360-degree laser retinopexy as the dependent variable to identify baseline characteristics associated with its use. Given the large number of independent variables (which reduces statistical power), *P* values < 0.10 were considered statistically significant in the first pass. To allow for inclusion in the model, PVR grades CP1-3, CP4-6, CP7-9 and CP10-12 were considered as one entity. A step-down elimination procedure was then used to obtain the final multiple logistic regression model.

The use of 360-degree laser retinopexy was also analysed for any association with the subsequent surgical decisions of scleral buckling and tamponade agent. Fisher’s exact test was used for PPV/PPV + SB and Chi-squared test for tamponade agent. A *P* value < 0.05 was considered to be statistically significant. Univariate logistic regression was used to compare the primary outcome of SSAS between patients with 360-degree laser and limited laser.

Given the significant difference in baseline variables and subsequent surgical choices between the study groups, the primary analysis was extended to multiple logistic regression for the dependent variable SSAS with independent variables selected based on significance in previous models. Again, a significance threshold of p < 0.10 was used for this regression and a step-down elimination procedure was used to obtain the final model.

Multivariate regression was used for the secondary outcomes of ERM development, postoperative CMO, final logMAR visual acuity, improvement in logMAR visual acuity and tonic pupil. Logistic regression was used for binary variables, and linear regression was used for continuous variables. In regression models, categorical variables were encoded as dummy variables. All statistical analysis was performed with Stata IC version 16.1 software.

## Results

### Patient characteristics

A total of 192 surgeries were included in the study; of which 130 had 360-degree laser retinopexy performed and 62 has limited laser retinopexy performed. The average patient age at the time of surgery was 63.6 ± 12.8. A summary of basic demographic and clinical variables is presented in Table 1.

**Table 1:** Baseline characteristics of patients undergoing PPV or combined PPV with SB

	360 Laser (n = 130)	Limited laser (n = 62)	*P* value
Age, mean (SD), years	63.2 ± 13.0	64.6 ± 12.3	0.087
Female, n (%)	31 (23.8%)	25 (40.3%)	0.069
Right eye	63 (48.5%)	33 (53.2%)	0.317
Preoperative logMAR visual acuity	1.6 ± 1.2 (~ 6/240)	1.1 ± 1.0 (~ 6/75)	0.017
Lens status			
Phakic (base variable)	77 (59.2%)	38 (61.3%)	
Pseudophakic	52 (40%)	23 (37.1%)	0.559
Aphakic	1 (0.8%)	1 (1.6%)	0.711
Macula status			
On (base variable)	36 (27.7%)	26 (41.9%)	
Off	91 (70.0%)	31 (50.0%)	0.763
Split/threatened	3 (2.3%)	5 (8.1%)	0.086
PVR grade			
Nil (base variable)	90 (69.2%)	50 (80.6%)	
A	3 (2.3%)	10 (16.1%)	0.013
B	18 (13.8%)	1 (1.6%)	0.022
CP1-3	9 (6.9%)	1 (1.6%)	0.377*
CP4-6	6 (4.6%)	0 (0.0%)	
CP7-9	2 (1.5%)	0 (0.0%)	
CP10-12	2 (1.5%)	0 (0.0%)	

^*^For statistical analysis, CP1-3, CP4-6, CP7-9 and CP10-12 were combined as ‘CP1-12’

### Patient factors associated with the use of 360-degree retinopexy

Multiple logistic regression was used to predict the use of 360-degree vs limited laser from preoperative logMAR visual acuity, sex and grade of PVR. In summary, worse preoperative logMAR visual acuity, male sex and higher PVR grades are significantly associated with the use of 360-degree laser retinopexy. These variables statistically significantly predicted the use of 360-degree laser, LR X^2^(5) = 39.72, p < 0.0001, pseudo R^2^ = 0.1644. Table 2 breaks down the results of the multiple regression for each variable.

**Table 2:** Multivariate regression analysis of the use of 360-degree laser retinopexy

	Odds ratio	95% CI	*P *value
Preoperative logMAR visual acuity	1.487	1.105 to 2.002	0.009
Female	1.981	0.971 to 4.042	0.060
PVR grade (base variable nil)			
A	0.150	0.038 to 0.602	0.007
B	9.670	1.238 to 75.522	0.030
CP1-12	7.429	0.943 to 58.494	0.057

### Surgical decisions associated with the use of 360-degree retinopexy

Table 3 presents the differences in scleral buckling and tamponade agents between the 360-degree laser and limited laser groups. Of the 45 patients who underwent scleral buckling in combination with PPV, 36 (80%) also received 360-degree laser. This resulted in a significant difference (*P* = 0.0468). There was also a significant difference (*P* =  < 0.0001) in tamponade agents, whereby limited laser patients were more likely to receive sulphur hexafluoride (SF_6_) and 360-degree laser patients were more likely to receive perfluoropropane (C_3_F_8_) and particularly silicone oil.

**Table 3:** Surgical decisions associated with the use of 360-degree laser retinopexy

	360 Laser (n = 130)	Limited laser (n = 62)	*P* value
Surgical technique			
PPV	94 (72.3%)	53 (85.5%)	0.0468
PPV with SB	36 (27.7%)	9 (14.5%)	
Tamponade agent			
SF_6_	15 (11.5%)	30 (48.4%)	< 0.0001
C_3_F_8_	65 (50.0%)	25 (40.3%)	
Silicone oil	50 (38.5%)	6 (9.7%)	

### Primary and secondary outcomes

The single surgery anatomical success rate in patients having undergone 360-degree laser retinopexy was 86.2% compared with 75.8% in patients who received limited laser retinopexy. This difference approached but did not reach statistical significance (*P* = 0.079) with univariate logistic regression. Table 4 displays a summary of all primary and secondary outcome variables. There was no statistical difference for any of the secondary outcomes when comparing the 360-degree laser and limited laser groups. Only one patient from either group had a documented corneal epithelial defect so this was excluded from the analysis. There was also no statistical difference (*P* = 0.2308) between the 360-degree laser and limited laser groups when analysing the visual acuity improvement from preoperative logMAR to final logMAR.

**Table 4:** Primary and secondary outcomes of patients undergoing PPV or combined PPV with SB

	360 Laser (n = 130)	Limited laser (n = 62)	*P* value
Single surgery anatomical success	112 (86.2%)	47 (75.8%)	0.079
Final logMAR visual acuity	0.9 ± 0.9 (~ 6/48)	0.6 ± 0.8 (~ 6/24)	0.0623
ERM development	17 (13.1%)	9 (14.5%)	0.8208
Postoperative CMO	11 (8.5%)	7 (11.3%)	0.5946
Tonic pupil	3 (2.3%)	1 (1.6%)	> 0.9999
Corneal epithelial defect	1 (0.8%)	0 (0.0%)	N/A

Taking into consideration the other variables identified as significantly different between the limited and 360-degree laser groups, multiple logistic regression was performed with SSAS as the dependent variable and choice of laser, tamponade agent, scleral buckling, preoperative LogMAR, sex and PVR as independent variables. Given that a difference in choice of laser intraoperatively was associated with differences in the other surgical factors of tamponade agent and the use of a scleral buckle, the interaction of these variables was included in the multiple regression analysis, and they were not analysed independently. These variables did not statistically significantly predict SSAS, LR X^2^(13) = 9.48, p < 0.7355, pseudo R^2^ = 0.0544. None of the variables examined were statistically significant (p > 0.01).

## Conclusions

Retinal re-attachment and prevention of re-detachment is necessary in RRD surgery to provide anatomical and functional improvement. The application of 360-degree laser retinopexy during primary PPV with or without SB has historically been advocated by some surgeons for detachments associated with giant retinal tears [15] or in cases where no retinal breaks can be identified [16]. Increasingly, surgeons have adapted this technique to routine RRD cases in the hopes of improving surgical outcomes. However, the factors associated with its use and the impact on surgical outcomes is not well understood, with conflicting reports in the literature. The theoretical benefit of applying 360-degree laser retinopexy is to seal any missed breaks and barricade any potential anterior re-detachment of the retina [4, 9]. However, this must be weighed against the theoretical increased risk of ERM development, postoperative CMO and new tears at laser boundaries.

In the current study, worse preoperative logMAR visual acuity, male sex and higher PVR grades were found to be significantly associated with the use of 360-degree laser retinopexy. PVR is a major risk factor for surgical failure following PPV for primary retinal detachment. Wickham et al. [8] found that grade C PVR was significantly associated with higher re-detachment rates and the presence of PVR at presentation resulted in a difference in the median vision of 2 logMAR lines at 6 months postoperatively. Our findings suggest that surgeons at the RBWH are more likely to implement 360-degree laser retinopexy in more complex cases with worse functional baseline and prognostic factors. A large, multicentre, retrospective study by Wang et al. [14] found that younger patient age, larger extent of retinal detachment, larger number of retinal breaks and surgeon preference were significantly associated with the application of 360-degree laser retinopexy. However, a number of smaller studies into the use of 360-degree laser retinopexy in RRD patients found no difference in the baseline characteristics of their groups [4, 10, 13, 17]. Patients with higher grades of PVR, a factor found to be significant in the current study, were excluded in some of these study designs. The variables of retinal detachment extent, number of retinal breaks and surgeon ID, all found to be significantly associated with 360-degree laser use by Wang et al. [14], were not reported in the current study. The field of RRD research may benefit from a standardisation of documented preoperative variables to allow for better understanding of factors impacts surgical decision making and reduce the risk of confounding variables.

The application of 360-degree laser retinopexy was significantly associated with the use of C_3_F_8_ and silicone oil as tamponade agents, and SB as a supplement to PPV. This further suggests that the decision to use 360-degree laser retinopexy was made in the context of primary RRD case complexity; with 360-degree laser more likely to be used as part of a more extensive operation. C_3_F_8_ and silicone oil have visual and anatomic advantages over SF_6_ in complex RD cases with PVR [18–20]. However, these agents impair vision longer which may delay or compromise a patient’s return to daily function. C_3_F_8_ also prohibits air travel for as long as 8 weeks whilst silicone oil requires follow-up surgery to remove the oil. In simpler cases requiring less tamponade, SF_6_ is an appropriate agent, allowing quicker visual rehabilitation for the patient. The decision of which patients may benefit from SB in combination with PPV for primary RRD repair is largely driven by preoperative exam findings and surgeon preference. Factors traditionally associated with higher risk of surgical failure have been used to justify SB at the time of PPV, including, pre-existing PVR, larger detachment size, inferior breaks, extensive lattice degeneration and phakic lens status [21]. However, there remains controversy in the literature as to whether supplemental SB offers any benefit over PPV alone in these cases [14, 21].

This retrospective cohort study included 192 consecutive patients who underwent PPV with or without SB at a single institution. The difference in SSAS (86.2% for 360-degree and 75.8% of limited) did not meet statistical significance, either outright or after accounting for confounding patient factors and surgical decisions with multiple logistic regression. There was no significant difference in final logMAR visual acuity. Despite 96.7% eyes having PVR less than Grade B in the limited laser group, the lower SSAS were most likely due to common causes of failure like missed breaks or PVR development. Although analysis of this is beyond the scope of this article, there was a higher use of SF6 in the limited group and tamponade choice may also have been be a factor in these patients.

A recent systematic review and meta-analysis by Soekamto et al. [9] found that prophylactic treatment with 360-degree laser had no significant effect on the initial rate of retinal re-detachment or final best-corrected visual acuity following PPV repair of RRD. Subgroup analysis of the six included studies found patients undergoing 23-gauge PPV alone had significantly higher attachment rates upon application of 360-degree laser when compared to limited laser. However, this result was largely due to Dirani et al. [10] (n = 151) who found 360-degree laser to be associated with a 75% reduction in the odds of retinal detachment. Bilgin et al. [4] (n = 50) and Loiudice et al. [13] (n = 93) both used 23-gauge PPV and did not find a statistical difference in SSAS between 360-degree and limited laser groups, however rates were higher in the 360-degree laser group in both studies. Falkner-Radler et al. [11] (n = 60) and Iwase [12] (n = 378) both used 20-gauge instruments and did not find 360-degree laser retinopexy to significantly impact SSAS in primary RRD patients. Wang et al. [14] (n = 2248) included 20-gauge (n = 104), 23-gauge (n = 1459), 25-gauge (n = 672) and 27-gauge (n = 8) in their study. They found no statistical difference in SSAS but found 360-degree laser to be significantly associated with lower final anatomical success and lower final logMAR visual acuity. No subgroup analysis was performed for different vitrectomy gauges. In the current study, all operations were performed with 25-gauge instruments. Our finding of no significant differences in surgical outcomes between 360-degree and limited laser retinopexy is incongruent with the hypothesis made by Soekamto et al. [9] that application of 360-degree laser plays a protective role following primary RRD repair using smaller gauge vitrectomy instruments.

ERM development [22], postoperative CMO [22], postoperative tonic pupil [23] and loss of corneal sensitivity with risk of neurotrophic ulcers [24] are reported complications of endolaser application during PPV. It is theorised that increased endolaser application during 360-degree laser retinopexy results in more inflammation and higher risk of developing these complications. The current study found no significant difference between the 360-laser and limited laser groups for ERM development (13.1% vs. 14.5%), postoperative CMO (8.5% vs. 11.3%), tonic pupil (2.3% vs. 1.6%) or corneal epithelial defects (0.8% vs. 0%). Bilgin et al. [4], Dirani et al. [10], Iwase et al. [12] and Wang et al. [14] also reported no significant difference in ERM development, with reported incidence ranging from 3.7% to 34.5%. No significant difference in CMO development was reported by Iwase et al. [12], Loiudice et al. [13] or Wang et al. [14], with reported incidence ranging from 0.7% to 8.6%. No other paper commented on tonic pupil or corneal epithelial defects, likely due to the rarity of these complications.

There remains controversy in the literature as to the impact of 360-degree laser retinopexy during PPV on surgical outcomes for primary RRD repair. The current study, along with many other studies on the topic, is limited by its relatively small patient population; reducing statistical power and ability to perform meaningful subgroup analysis. Our study is also retrospective, meaning that baseline patient characteristics and associated surgical decisions were not standardised. More complex cases and more extensive operations were seen in the 360-degree laser group. This was mitigated as best as possible with multiple logistic regression analysis. We recommend that a randomised, prospective, large-scale clinical trial with subsequent subgroup analysis is performed to definitively determine the anatomical and function efficacy, complication rates and best indication for 360-degree laser retinopexy during PPV for primary RRD repair.

In conclusion, we found worse preoperative logMAR visual acuity, male sex and higher PVR grades to be significantly associated with the use of 360-degree laser retinopexy during PPV with or without SB for primary RRD repair. 360-degree laser retinopexy was more likely to be applied in more extensive operations, with significant association with C_3_F_8_ and silicone oil tamponade agents and supplemental SB. Even when accounting for these factors, there is no significant difference in SSAS, final logMAR visual acuity, ERM development, postoperative CMO, tonic pupil or corneal epithelial defects between 360-laser and limited laser groups.

## Data Availability

The datasets used and/or analysed during the current study are available from the corresponding author on reasonable request.
